# A Rare Presentation of Guillain-Barré Syndrome With Associated Horner Syndrome: A Case Report

**DOI:** 10.7759/cureus.57188

**Published:** 2024-03-29

**Authors:** Shahul Irfan, Jayashree Ganesan, Kashish V Jain, Garvin Ahalya A P, Umarani Ravichandran

**Affiliations:** 1 Internal Medicine, Government Medical College & Hospital Cuddalore, Chidambaram, IND

**Keywords:** guillain-barré syndrome (gbs), intravenous immunoglobulin (ivig), unilateral ptosis, immunemediated paraparesis, rare variant, horner’s syndrome

## Abstract

Guillain-Barré syndrome (GBS) is an acute inflammatory polyradiculoneuropathy involving the peripheral nervous system. Autonomic dysfunctions are well-known complications of GBS and are major contributors to mortality. Autonomic dysfunctions are classically described during the acute phase of illness. In the literature, Horner syndrome as a manifestation of GBS has been reported in very few cases. Here, we describe a case of GBS with an acute presentation of flaccid paraparesis associated with unilateral Horner syndrome. Detecting the cause of acute flaccid paraparesis with unilateral Horner syndrome poses a diagnostic challenge, making it crucial for clinicians to maintain a heightened awareness for distinguishing between GBS and its variants, as well as other potential mimics.

## Introduction

Guillain-Barré syndrome (GBS) is an immune-mediated disorder with an incidence of 0.4-2.4/100,000 population annually, having a slight preponderance of young adults over the elderly and mortality of about 3-10% [1]. This disorder exhibits heterogeneity with numerous variants, each characterized by unique features. GBS subtypes are classified according to nerve conduction studies, with acute inflammatory demyelinating polyneuropathy (AIDP) being the most common type characterized by demyelinating pathology, while acute motor axonal neuropathy (AMAN), acute motor-sensory axonal neuropathy (AMSAN), and Miller-Fisher syndrome involve axonal loss, leading to axonal neuropathies [2]. Certain individuals present with unique clinical manifestations of GBS that deviate from the typical pattern of sensory loss and weakness. Some of the common atypical variants include pure motor variant, bilateral facial palsy with paresthesia, pharyngeal-cervical-brachial weakness, and paraparetic variant [3-5]. Despite the distinct features of each variant, they may also exhibit atypical characteristics, posing a diagnostic challenge for physicians when patients present with them. Autonomic involvement in GBS necessitates prompt recognition and management to prevent life-threatening complications. Horner syndrome in GBS is believed to be due to immune-mediated damage to the cervical sympathetic nerve fibers that innervate the pupil, eyelid, and sweat glands of one-half of the face [6].

## Case presentation

History

Our case is a 30-year-old woman who was admitted to the neurology department with five days' history of pain and numbness of bilateral lower limbs associated with weakness. She was apparently normal before the five days, after which she felt a deep boring pain in her knees. Then, she developed numbness in her feet on the second day of illness associated with weakness in her legs. The weakness was gradually progressive, and at the time of admission to the hospital, she developed weakness in both proximal and distal muscles of her lower limbs. She also noted drooping of the right eyelid on day three of her illness. She did not complain of any double vision and diurnal variation in the drooping of her eyelid. She exhibited no signs of respiratory difficulty, swallowing issues, or bowel and bladder incontinence. She denied a history of preceding gastrointestinal, respiratory, or other infections. In addition, she reported no alcohol consumption, smoking habits, or any recent vaccinations.

Nervous system examination

**Figure 1:**
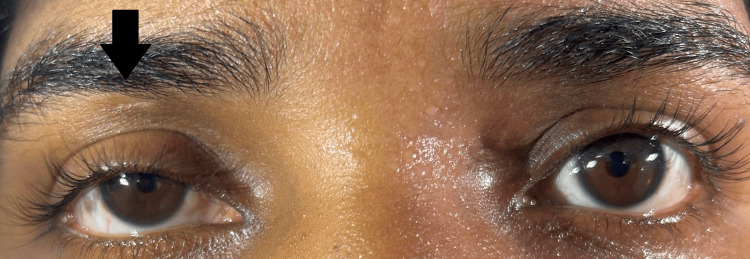
There is a partial ptosis in the right eyelid with a miotic pupil and a normal left eye.

**Figure 2:**
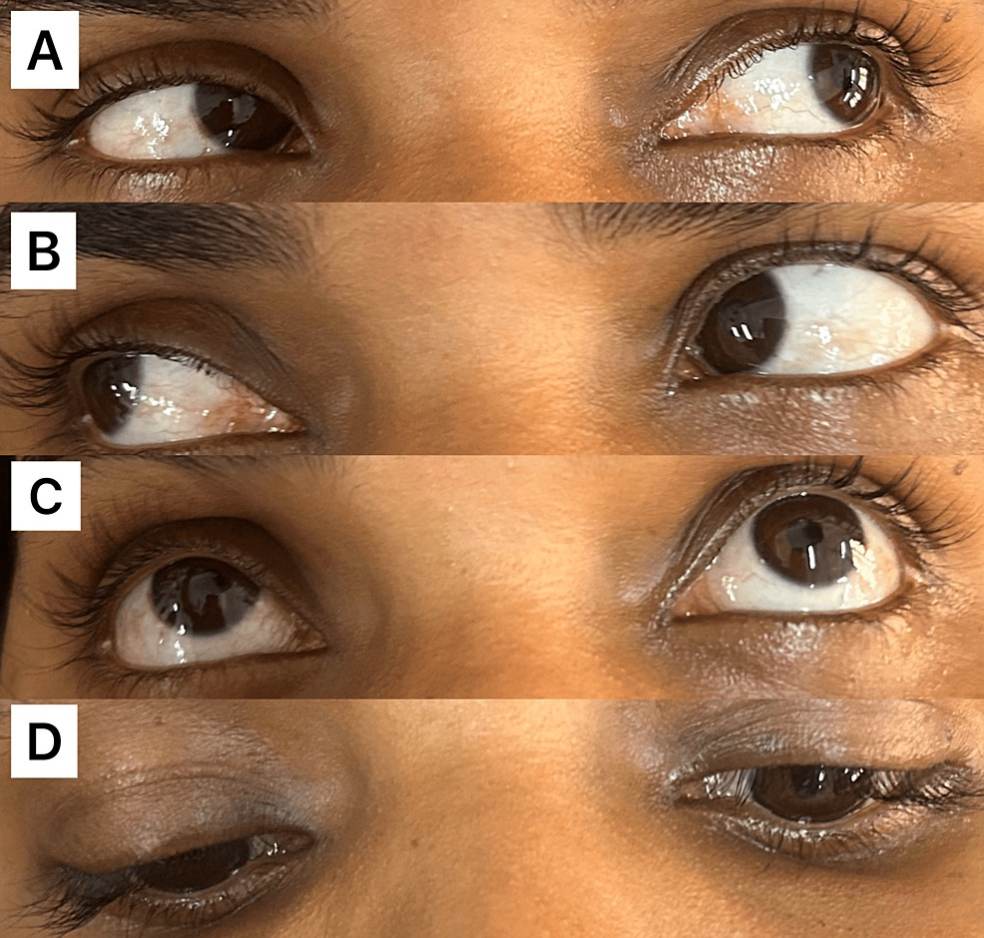
Extraocular movements of the patient. Figure 2 (A-D) shows that the patient's extraocular movements are full, with no signs of ophthalmoplegia.

**Figure 3:**
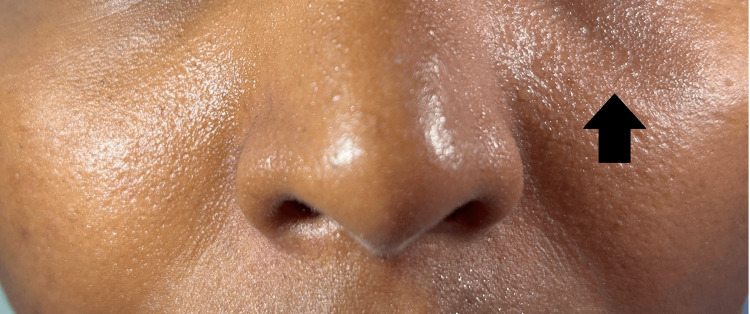
Sweating is present on the left cheek and left upper lip with the absence of sweating on the right side.

Examination of her motor system revealed, decreased power in both proximal and distal muscles of the lower limbs (grade 2/5) with no signs of weakness elsewhere. Her lower limbs were hypotonic with absent deep tendon reflexes (grade 0). Reflexes of the upper limbs were diminished (grade 1). Examination of the sensory system revealed a decreased sensation on her legs, extending up to the knees with an absent plantar response. Ocular examination revealed a partial ptosis on her right eye and a miotic pupil with a pupillary diameter of 1 mm (Figure 1). Her eyelid and pupil on the left side were normal, and there were no signs of ophthalmoplegia (Figure 2A-2D). Pupillary light and accommodation reflex were normal in both eyes. She also had unilateral dryness of her face on the right side, accompanied by the absence of sweating (Figure 3).

Examinations of the cranial nerves, higher mental function, and cerebellum showed no abnormalities. Examination of the autonomic nervous system including the test for postural hypotension (tilt table test), the Valsalva maneuver, and baroreflex sensitivity measurement yielded normal results, with the exception of notable findings in the eye and face.

Investigations and treatment

**Figure 4:**
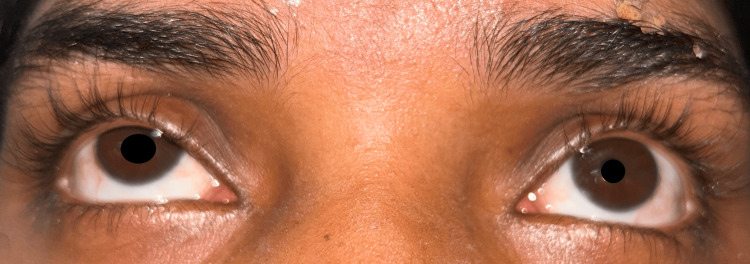
There is a partial resolution of the ptosis and dilation of the right pupil, after application of 0.5% Apraclonidine.

Her lab investigations, including full blood count, erythrocyte sedimentation rate, C-reactive protein, liver function, renal function, and serum potassium, magnesium, and calcium levels, yielded normal results. A nerve conduction study showed severe demyelinating type of polyneuropathy in the lower limbs. There was no decremental response to repetitive nerve stimulation. The ice-pack test failed to improve the patient’s ptosis. Application of topical Apraclonidine (0.5%) on her eyes led to dilation of the right pupil (2 mm) and resolution of the right ptosis with no significant changes in the left eye (Figure 4).

MRI scan of the brain, whole spine, neck, and CT thorax showed no significant abnormality. Serum antibody panel for GBS including anti-GM1, anti-GD1a, and anti-GQ1b antibodies were negative. A provisional diagnosis of acute flaccid paraparesis with an associated Horner syndrome, most likely attributable to GBS, was made. She was started on intravenous immunoglobulin (IVIg) with a standard dose of 0.4 g/kg/day for five consecutive days (total dose of 2 gm/kg body weight). A lumbar puncture done on the 10th day of her illness showed an elevated CSF protein of 512 mg/dL with no white cells. CSF cultures and gram staining yielded negative results. The presence of cell protein dissociation and negative cultures supported the diagnosis of GBS. She was meticulously monitored in the ICU for two weeks with daily assessments, including tidal volume measurements, blood pressure, heart rate, and nervous system examinations. There was no further deterioration in the patient’s clinical condition. Two weeks after completion of IVIg treatment, a gradual improvement in her ptosis and miotic pupil was observed. On follow-up for one month, she had a complete recovery of her ocular signs, facial anhidrosis, and lower limb numbness with a partial recovery of her weakness (improved from grade 2 to grade 4). She was given physiotherapy and discharged from the hospital after a month and regularly followed up in the clinic for six months. Despite adequate physiotherapy, the power in the proximal and distal muscles of her lower limbs did not improve further.

## Discussion

GBS is the most common cause of acute flaccid paralysis in clinical practice. The usual pattern is an ascending paralysis with weakness typically evolving over hours to a few days [7]. It is caused by an aberrant cell-mediated and humoral immune response (molecular mimicry) to infectious agents or vaccines, resulting in damage to the peripheral nerves. The most common organisms associated with GBS are *Campylobacter jejuni* (most common), Cytomegalovirus, hepatitis E virus, *Mycoplasma pneumonia*, Epstein-Barr virus, and Zika virus [8]. Not all patients with GBS necessarily experience a preceding infection, highlighting the variability in its etiology and clinical presentation. Infiltration of macrophages and complement activation are typical characteristics of GBS. The neural targets are gangliosides, which play a crucial role in cell-cell interactions and regulation of growth of the nervous tissue. They reside in high densities in the myelin sheaths and nodes of Ranvier [9]. The present case is a good example of an atypical paraparetic variant with associated Horner syndrome. Autonomic nervous system involvement in GBS occurs in almost one-third of patients. A study conducted by Singh et al. found that 41.53% of GBS patients experienced autonomic dysfunction, with constipation (22%) and diarrhea (21.2%) being the most prevalent manifestations [10]. Other observed symptoms comprised urinary retention (15.3%) and fluctuations in blood pressure and heart rate, each reported at 13.6% [10]. 

Horner syndrome, a rare condition, typically manifests with partial ptosis (drooping of the upper eyelid), miosis (constriction of the pupil), and facial anhidrosis (lack of sweating) as a result of disruption in sympathetic nerve supply [6]. The superior tarsal muscle, innervated by sympathetic nerves, assists in lifting the upper eyelid. Denervation of this muscle in Horner syndrome causes partial ptosis, milder than oculomotor nerve palsy [6]. The absence of ophthalmoplegia also rules out oculomotor nerve palsy as the cause of ptosis. Horner syndrome as the cause of partial ptosis and miosis can be confirmed by topical Apraclonidine test. In Horner syndrome, the affected pupil and the tarsal muscle experience denervation hypersensitivity. Apraclonidine, an alpha-2 adrenergic agonist with weak alpha-1 adrenergic agonist activity, when applied topically, induces dilation of the affected pupil and potentially elevates the lid, while leaving the normal pupil unchanged [11]. Horner syndrome predominantly arises as an acquired condition, often stemming from systemic or local diseases or iatrogenic causes. Regarding differential diagnosis of this case, the secondary causes of Horner syndrome, including central nervous system infections (meningitis, meningoencephalitis), demyelinating conditions (multiple sclerosis), vascular causes (lateral medullary syndrome), CNS malignancies, and local causes including cervical rib, mediastinal lymph nodes, and Pancoast tumor, were ruled out by appropriate clinical examination and radiological investigations. The motor, sensory, and autonomic manifestations in GBS are typically bilateral but can also be asymmetrical [7]. In the present case, there was asymmetrical involvement of the cervical sympathetic chain causing unilateral Horner syndrome.

The clinical course of GBS is usually monophasic with an initial progressive phase for two weeks followed by a plateau phase that can last from weeks to months, after which recovery sets in. About 60-80% of patients are able to walk independently after six months of disease onset with or without treatment [12]. Treatment of GBS should commence upon meeting any of the following criteria: inability to walk independently for more than 10 meters, rapid progression of weakness, severe autonomic dysfunction or swallowing difficulty, or respiratory insufficiency [5]. The management options include intravenous immunoglobulin (IVIg) at a dose of 0.4 g/kg body weight/day for five days or plasma exchange at a rate of 250 ml plasma/kg body weight in five sessions. Both regimens are equally effective in managing GBS. IVIg is usually the treatment of choice as it is easier to administer and more widely available [13]. Besides IVIg and plasma exchange, no other treatment has been proven effective in managing GBS. Relapses in GBS are known to occur in 2-5% of patients [14]. Complications of GBS include hospital-acquired infections, cardiac arrhythmias, paralytic ileus, aspiration pneumonia, hyponatremia, and compression neuropathies [5]. Careful monitoring of patients during the course of illness is inevitable in managing and preventing complications.

## Conclusions

GBS is a complex clinical syndrome that frequently involves an acute inflammatory polyradiculoneuropathy affecting the peripheral nervous system. Autonomic dysfunction, which is well-known as one of the complications of GBS, contributes significantly to its high mortality, especially during the acute stages of the disease. The present case is a rare case of a paraparetic variant of GBS with associated unilateral Horner syndrome and responded well to IVIg treatment. Although Horner syndrome in relation to GBS is uncommon, our case underlines the need for the recognition and appropriate management of unusual presentations. The diagnostic challenge posed by acute flaccid paraparesis with unilateral Horner syndrome accentuates clinicians' responsibility to be vigilant in differentiating between GBS and its variants, as well as other possible mimics. Effective management of GBS relies on prompt interventions, guided by established criteria for treatment initiation. Despite being monophasic in nature, GBS necessitates close monitoring and comprehensive care planning aimed at reducing complications while optimizing patient outcomes. In view of ongoing developments in our understanding of GBS, continuous research endeavors and clinical alertness remain fundamental toward improving our diagnostic and management strategies that will ultimately enhance results for patients.
